# The impact of the UK soft drink industry levy on ethnic inequalities in admission rates for caries-related extractions

**DOI:** 10.1093/pubmed/fdag016

**Published:** 2026-02-21

**Authors:** C C Salomon-Ibarra, J Wu, V Toffolutti, E Bernabe

**Affiliations:** Institute of Dentistry, Queen Mary University of London, Turner Street, London E1 2AD, London, UK; Wolfson Institute of Population Health, Queen Mary University of London, 58 Turner Street London E1 2AB, London, UK; Wolfson Institute of Population Health, Queen Mary University of London, 58 Turner Street London E1 2AB, London, UK; Institute of Dentistry, Queen Mary University of London, Turner Street, London E1 2AD, London, UK

**Keywords:** dental care for children, dental caries, health inequalities, sugar-sweetened beverages, taxes, tooth extraction

## Abstract

**Objective:**

To assess the impact of the soft drinks industry levy (SDIL) on ethnic inequalities in hospital admissions for caries-related extractions among children in England.

**Methods:**

NHS hospital admission rates for caries-related extractions among 0–17-year-olds, between March 2007 and December 2024, were stratified into five ethnic groups (white, black, South Asian, mixed and others). Absolute and relative inequalities in admission rates were assessed using the weighted mean difference from the reference group (WMDR) and the Theil index, respectively. The impact of the SDIL on each ethnic inequality measure was quantified in a segmented regression model against a counterfactual scenario of no implementation.

**Results:**

Compared to the counterfactual scenario of no implementation, there were no absolute reductions in the WMDR at 22 months (0.04, 95% CI: −0.58 to 0.65) and 80 months (−0.16, 95% CI: −1.21 to 0.88) after implementation. Similarly, there were no absolute reductions in the Theil index at 22 months (−0.66, 95% CI: −8.01 to 6.68) and 80 months (−6.15, 95% CI: −19.36 to 7.07).

**Conclusions:**

The introduction of the SDIL was not associated with reductions in ethnic inequalities in admission rates for caries-related extractions.

## Introduction

An umbrella review on the effect of sugar-sweetened beverages (SSBs) taxation on dental caries suggested a 20% volumetric SSB tax would have a modest impact on caries prevalence and severity in high-income and low-and-middle-income countries.^1^ Empirical evaluations of the effect of SSB taxes on dental caries are limited: a study from Mexico showed the tax was associated with reductions in caries-related outcomes,^2^ while a study from the USA found no overall impact.^3^

In England, the soft drinks industry levy (SDIL) was announced in March 2016 and implemented in April 2018. It applies a two-tier levy: drinks with 8 g of sugar or more per 100 mL are taxed at 24 pence per litre, and drinks with 5–7.9 g at 18 pence per litre. Drinks under 5 g per 100 mL are exempt.^4^ The introduction of the SDIL was linked to lower admission rates for caries-related extractions and reduced deprivation-related inequalities in children.^5^^,^^6^ Assessing the SDIL’s impact on health inequalities across other social factors, such as race and ethnicity, remains essential to promote equitable benefits.

Ethnic inequalities in childhood dental caries in England are multifaceted. National data indicate clear differences at ages 5 and 8 years, with Asian children having more decayed teeth than white children, while black, and other ethnic minority groups had fewer decayed teeth. By ages 12 and 15, however, these ethnic differences in decayed teeth were minimal.^7^ Given the established causal link between sugars intake and dental caries,^8^ it is plausible that measures to reduce SSB consumption, such as the SDIL, could help narrow inequalities in dental caries. The mechanism may operate through differential baseline consumption assuming some ethnic groups consume more SSBs. However, the limited evidence on ethnic differences in SSB consumption is inconsistent. While one study reported higher SSB consumption among Asian than white children, but not among black children,^9^ another study reported no differences in consumption of soft drinks and 100% fruit juices between white and non-white children.^10^ The present study adds to current knowledge by assessing the impact of the SDIL on absolute and relative ethnic inequalities in hospital admissions for caries-related extractions among children in England. Admissions for caries-related dental extractions are widely accepted as a robust, population-level marker of the most severe end of the dental caries distribution in children, reflecting both high disease burden and failure of preventive care.^11^^,^^12^

## Methods

A natural experimental design with a controlled interrupted time series (ITS) analysis was used to assess observed changes following the implementation of the SDIL against a counterfactual scenario of no implementation. The study follows recommendations for the design and analysis of ITS,^13^^,^^14^ and the REporting of studies Conducted using Observational Routinely-collected health Data (RECORD) guidelines.^15^ The protocol registration identifier is ISRCTN17412377.

### Data source

This study used monthly data on hospital admissions in NHS hospitals in England, between April 2007 and December 2024 for 0–17-year-olds, from the Hospital Episode Statistics (HES) Admitted Patient Care database.^16^ Anonymised data were accessed through NHS England’s Secure Data Environment (SDE) after approval of a Data Access Request Service (DARS) application. Data were extracted in aggregated form, with monthly counts rounded to the nearest increment of 5 for numbers above 10 or suppressed for numbers below 10. The aggregated data included 212 months (107 before SDIL announcement, 25 between announcement and implementation and 80 after implementation).

### Outcome data

The primary outcome was hospital admissions for extraction of carious teeth. Consistent with the method used in government reports,^17^ they were defined as all finished consultant episodes with a primary procedure (OPSC-4) code for surgical removal of tooth (F09) or simple extraction of tooth (F10) and a primary diagnosis (ICD-10) code for dental caries (K02.1, K02.5, K02.8, and K02.9) or diseases of pulp and periapical tissues (K04.0, K04.5, K04.6, and K04.7). Admissions for tonsillectomies (F34.1 to F34.9) were selected as a negative control outcome (e.g. an outcome that should not be impacted by the SDIL) because they are a common reason for hospitalisation among children which were also affected by the lockdowns during the COVID-19 pandemic.

Standardised monthly hospital admissions rates were produced by dividing the number of hospital admissions by the estimated population size for each month and multiplying by 100 000. Mid-year population estimates for children under 18 years old, disaggregated by ethnicity, were obtained from census data published by the Office for National Statistics. For non-census years, estimates for each ethnic group were extrapolated by assuming linear growth between 2001, 2011, and 2021 census years, ensuring consistency with total mid-year population estimates (0–17-year-olds) for each year.

Aggregated hospital admission counts were obtained for five ethnic groups: South Asian, black, white, mixed, and other. Table S1 shows how available categories were combined into five ethnic groups.^18^ HES records with non-stated/non-recorded ethnicity (18.6% for caries-related extractions and 14.2% for tonsillectomies) were excluded as no population denominators were available to calculate rates.

The magnitude of absolute and relative inequalities in admission rates was assessed using the weighted mean difference from the reference group (WMDR) and the Theil index, respectively. The WMDR measures the average absolute difference between each ethnic group’s rate and that of the reference group (white group), with each difference weighted by the group’s population share. The Theil index is calculated by comparing each ethnic group’s share of admission rates to their share of the population, using logarithms to capture differences. In both health inequality measures, a value of zero indicates no differences among ethnic groups, and therefore perfect equality. Values greater than zero suggest the presence of inequalities in admission rates between ethnic groups.^19^

### Statistical analysis

ITS analysis was used to assess the impact of the SDIL on WMDR and Theil index for the primary and negative control outcomes. A key analytical challenge was to disentangle the impact of SDIL from that of the COVID-19 lockdown restrictions. Hospital admissions decreased between March 2020 and July 2021,^20^^,^^21^ but gradually recovered six months after lockdowns ended.^22^ Another analytical challenge was to address the change in ethnicity coding in HES records introduced in April 2013. This change affected how ethnicities were coded (using numbers instead of letters from 2013) and the ethnic categories available. Thus, a segmented regression model was fitted with a parameterization (impact model) that incorporated six key policy and contextual phases, including the old ethnicity coding, new ethnicity coding, SDIL announcement, SDIL implementation, COVID-19 lockdown, and post-lockdown recovery periods.^23^^,^^24^ The model specification was as follows:


\begin{align*} {Y}_t=&\ {\beta}_0+{\beta}_1T+{\beta}_2C+{\beta}_3C{T}_C+{\beta}_4A+{\beta}_5A{T}_A\\&+{\beta}_6E+{\beta}_7E{T}_E+{\beta}_8L+{\beta}_9L{T}_L+{\beta}_{10}R\\&+{\beta}_{11}R{T}_R+{\in}_t \end{align*}

Where ${Y}_t$ is the ethnic inequality measure at month $t$, ${\beta}_0$ and ${\beta}_1$ are level (intercept) and trend (slope) before change in ethnicity coding and $T$ is time since the start of the series. $C$ is a dummy variable for *new ethnicity coding period* (0 before April 2013, 1 from April 2013 onwards), ${T}_c$ is time since change in ethnicity coding (0 up to and including April 2013, increasing by one each month thereafter), and ${\beta}_2$ and ${\beta}_3$ are immediate changes in level and trend after change in ethnicity coding. $A$ is a dummy variable for *announcement period* (0 before March 2016, 1 from March 2016 onwards), ${T}_A$ is time since announcement (0 up to and including March 2016, increasing by one each month thereafter), and ${\beta}_4$ and ${\beta}_5$ are immediate changes in level and trend after announcement (relative to previous segment). $E$ is a dummy variable for *implementation period* (0 before April 2018, 1 from April 2018 onwards), ${T}_E$ is time since implementation and ${\beta}_6$ and ${\beta}_7$ are immediate changes in level and trend after implementation (relative to previous segment). $L$ marks the *lockdown period* (0 before March 2020, 1 from March 2020 onwards), ${T}_L$ is time since start of lockdowns, and ${\beta}_8$ and ${\beta}_9$ are immediate changes in level and trend after lockdown began (relative to previous segment). $R$ is a dummy variable for *recovery (post-lockdown) period* (0 before January 2021, 1 from January 2022 onwards), ${T}_R$ is time since recovery; and ${\beta}_{10}$ and ${\beta}_{11}$ are immediate changes in level and trend after lockdowns ended (relative to previous segment). Using this formulation, the level and trend changes in announcement period were ${\beta}_4$ and ${\beta}_5$, those in implementation period were $({\beta}_4+{\beta}_6)$ and $({\beta}_5+{\beta}_7$), those in lockdown period were $({\beta}_4+{\beta}_6+{\beta}_8)$ and $({\beta}_5+{\beta}_7+{\beta}_9)$, and those in recovery period were $({\beta}_4+{\beta}_6+{\beta}_8+{\beta}_{10})$ and $({\beta}_5+{\beta}_7+{\beta}_9+{\beta}_{11})$, compared to level (${\beta}_2)$ and trend (${\beta}_3)$ in new ethnicity coding period.

To assess the SDIL’s impact, observed inequality measures were compared against a counterfactual scenario (the predicted trend had the pre-announcement data [new ethnicity coding period, April 2013 to February 2016] continued without intervention). Absolute and relative differences between observed and counterfactual values with 95% confidence intervals were estimated at 22- and 80-months post-implementation.^23^^,^^24^ These timepoints were pre-specified to capture short- and long-term impacts of the SDIL. The earlier point corresponds to the month before the first national lockdown (February 2020) while the latter point corresponds to the final month in the ITS (December 2024). For sensitivity analysis, all models were refitted using only the portion of the ITS after the change in ethnicity coding (from April 2013, 141 months). Analyses were performed in Stata (StataCorp, College Station, TX).

## Results

Compared with white children, admission rates for caries-related extractions were higher among Asian and other ethnic groups, but lower among black and mixed children (Table 1). Both WMDR and Theil index increased slightly after implementation of the SDIL but decreased during the recovery periods. Admission rates for tonsillectomies were higher among children of other ethnic groups but lower among Asian, black and mixed children compared with white children. Both WMDR and Theil index decreased during the implementation and recovery periods. Trends in admission rates for caries-related extractions and tonsillectomies according to ethnic groups are shown in Fig. S1.

**Table 1:** Mean and standard deviation (SD) for admission rates, absolute and relative inequalities for caries-related extractions and tonsillectomies.

	Old ethnicity coding period	New ethnicity coding period	Announcement period	Implementation period	Lockdown period	Recovery period
Number of months	72	35	25	23	22	36
Start date	Apr 2007	Apr 2013	Mar 2016	Apr 2018	Mar 2020	Jan 2022
End date	Mar 2013	Feb 2016	Mar 2018	Feb 2020	Dec 2021	Dec 2024
*Caries-related extractions*						
Missing data	14.69%	15.67%	19.51%	22.89%	23.76%	25.51%
Asian	30.19 (2.95)	29.99 (3.22)	22.93 (2.34)	19.05 (1.75)	10.39 (4.76)	16.42 (1.98)
Black	28.64 (4.48)	22.51 (3.99)	16.24 (2.51)	12.69 (2.26)	7.31 (3.37)	12.38 (2.08)
White	22.25 (2.36)	23.80 (2.11)	22.06 (1.79)	20.70 (1.91)	10.82 (4.34)	16.14 (2.06)
Mixed	16.98 (3.39)	18.02 (2.69)	15.13 (2.30)	12.71 (1.58)	6.98 (3.03)	10.51 (1.97)
Other	60.56 (8.75)	47.42 (5.80)	38.42 (3.60)	30.46 (4.05)	16.26 (6.88)	24.30 (4.42)
WMDR	1.93 (0.28)	1.66 (0.25)	1.34 (0.23)	1.44 (0.29)	0.79 (0.34)	1.04 (0.21)
Theil	23.15 (4.88)	14.28 (3.83)	12.64 (3.31)	14.22 (3.41)	14.04 (4.47)	12.54 (4.50)
*Tonsillectomies*						
Missing data	13.51%	12.58%	15.16%	15.65%	16.98%	15.09%
Asian	19.02 (2.99)	17.19 (2.53)	14.84 (1.78)	14.06 (2.04)	5.49 (2.79)	11.82 (3.17)
Black	18.66 (3.04)	17.40 (2.34)	14.10 (2.05)	14.65 (2.10)	5.71 (3.25)	12.03 (2.98)
White	20.32 (2.86)	21.21 (2.86)	19.23 (2.21)	17.47 (2.43)	8.02 (3.93)	14.69 (3.43)
Mixed	11.09 (2.12)	12.89 (1.88)	11.65 (1.72)	10.93 (1.57)	5.09 (3.08)	10.22 (2.44)
Other	29.20 (5.70)	31.17 (5.36)	23.56 (4.08)	19.85 (2.99)	9.39 (4.50)	15.77 (3.78)
WMDR	0.92 (0.25)	1.35 (0.32)	1.34 (0.37)	1.06 (0.33)	0.69 (0.33)	0.93 (0.28)
Theil	9.57 (3.33)	11.05 (3.32)	11.77 (4.58)	8.89 (3.71)	15.55 (7.10)	8.29 (4.10)

WMDR: Weighted mean difference from the reference group.

**Figure 1:**
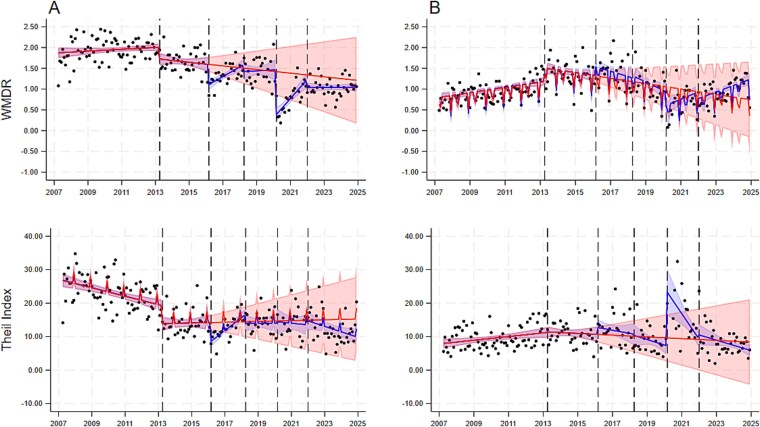
Absolute and relative ethnic inequalities in admission rates for caries-related extractions (panel A) and tonsillectomies (panel B) among 0–17-year-old children in England from April 2007 to December 2024. Absolute and relative inequalities were measured using the weighted mean difference from the reference group (WMDR) and the Theil index, respectively. The black dots show the observed data; the blue line indicates the predicted trend with 95%CI (blue shadow) fitted to the observed data and the red line indicates the counterfactual trend with 95%CI (red shadow) based on data from the pre-announcement period. The five dashed vertical lines indicate the dates of change in ethnicity code (April 2013), SDIL announcement (March 2016), SDIL enforcement (April 2018), and start (March 2020) and end of lockdowns (January 2022).

At the start of the new ethnicity coding period, the WMDR showed that caries-related extractions admission rates were, on average, 1.59 (95% CI: 1.35, 1.83) units higher in minority ethnic groups than the white group. However, the trend in WMDR was static (−0.004 [−0.01, 0.005]). Compared to these estimates, there were changes in level (−0.48 [−0.71, −0.26]) and trend (0.02 [0.01, 0.04]) in the announcement period; change in level (−0.65 [−1.02, −0.29]) but not in trend (0.01 [−0.01, 0.03]) in the implementation period; and change in level (−1.93, [−2.55, −1.31]) but not in trend (0.004, [−0.01, 0.01]) in the recovery period. The WMDR for tonsillectomy admission rates showed no changes in level or slope in the announcement, implementation or recovery periods, except for an increasing trend (widening of ethnic inequalities) in the recovery period (Table 2, Fig. 1). Comparing the observed trend (fitted values for the later phase) to the counterfactual (predicted trend from before announcement), there were no reductions in the WMDR for caries-related extraction admission rates 22 and 80 months after implementation. No differences were found for tonsillectomies either (Table 3).

**Table 2:** Models for the impact of the soft drinks industry levy (SDIL) on absolute and relative inequalities in admission rates for caries-related extractions and tonsillectomies.

	WMDR	[95% CI]	Theil	[95% CI]
*Caries-related extractions*				
New ethnicity coding period	1.59	[1.35, 1.83]	21.46	[17.51, 25.41]
Time since new ethnicity coding	−0.004	[−0.01, 0.005]	0.01	[−0.10, 0.12]
Announcement period	−0.48	[−0.71, −0.26]	−5.69	[−8.64, −2.74]
Time since announcement	0.02	[0.01, 0.04]	0.31	[0.15, 0.47]
Enforcement period	−0.65	[−1.02, −0.29]	−8.32	[−12.93, −3.71]
Time since implementation	0.01	[−0.01, 0.03]	−0.01	[−0.25, 0.23]
Lockdown period	−1.73	[−2.28, −1.19]	−7.89	[−15.22, −0.56]
Time since lockdowns began	0.04	[0.03, 0.06]	−0.05	[−0.34, 0.24]
Recovery period	−1.93	[−2.55, −1.31]	−6.69	[−15.71, 2.33]
Time since recovery	0.004	[−0.01, 0.01]	−0.15	[−0.32, 0.02]
*Tonsillectomies*				
New ethnicity coding period	1.11	[0.87, 1.36]	8.13	[5.12, 11.15]
Time since new ethnicity coding	0.00	[−0.01, 0.003]	−0.02	[−0.12, 0.07]
Announcement period	0.21	[−0.05, 0.47]	2.04	[−1.29, 5.37]
Time since announcement	−0.01	[−0.02, 0.01]	−0.06	[−0.27, 0.15]
Enforcement period	0.26	[−0.25, 0.77]	1.68	[−4.50, 7.86]
Time since implementation	−0.01	[−0.03, 0.01]	−0.12	[−0.37, 0.14]
Lockdown period	−0.07	[−0.74, 0.60]	17.69	[7.34, 28.03]
Time since lockdowns began	0.02	[0.0001, 0.04]	−0.62	[−1.02, −0.21]
Recovery period	−0.23	[−0.98, 0.53]	19.00	[7.14, 30.86]
Time since recovery	0.02	[0.01, 0.03]	−0.11	[−0.27, 0.05]

WMDR: Weighted mean difference from the reference group.

A value of zero in the WMDR and Theil index indicate no inequality. Values greater than zero indicate higher admission rates in minority ethnic groups than the white group (reference).

**Table 3:** Predicted differences between the fitted and counterfactual trends for absolute and relative inequalities in caries-related extractions and tonsillectomies 22 and 80 months after the SDIL.

	Absolute difference	[95% CI]	Relative difference (%)	[95% CI]
*WMDR*				
*Caries-related extractions*				
At 22 months	0.04	[−0.58, 0.65]	2.73	[−41.41, 46.86]
At 80 months	−0.16	[−1.21, 0.88]	−13.58	[−88.45, 61.29]
*Tonsillectomies*				
At 22 months	−0.11	[−0.64, 0.43]	−10.09	[−56.06, 35.88]
At 80 months	0.38	[−0.60, 1.37]	106.81	[−456.01, 669.62]
*Theil index*				
*Caries-related extractions*				
At 22 months	−0.66	[−8.01, 6.68]	−4.52	[−52.64, 43.60]
At 80 months	−6.15	[−19.36, 7.07]	−33.21	[−82.79, 16.37]
*Tonsillectomies*				
At 22 months	−2.30	[−9.70, 5.11]	−23.79	[−84.55, 36.97]
At 80 months	−2.43	[−15.28, 10.43]	−28.89	[−138.69, 80.91]

Differences were estimated by subtracting the predicted values for the fitted trend (using the full time series) from the counterfactual trend (based on pre-announcement data only).

In the new ethnicity coding period, the Theil index showed that caries-related extraction admission rates were 21.46 (17.51, 25.41) units higher in minority ethnic groups than the white group, although the trend for Theil index was static (0.01 [−0.10, 0.12]). Compared to these estimates, there was a decrease in level (−5.69 [−8.64, −2.74]) and increasing trend (0.31 [0.15, 0.47]) in the announcement period; a decrease in level (−8.32 [−12.93, −3.71]) but no change in trend (−0.01 [−0.25, 0.23]) in the implementation period; and no change in level (−6,69 [−15.71, 2.33]) or trend (−0.15 [−0.32, 0.02]) in the recovery period. The Theil index for tonsillectomy admission rates showed no changes in level or slope in the announcement, implementation or recovery periods, except for an increase in level (widening of ethnic inequalities) in the recovery period (Table 2, Fig. 1). Comparing the observed and counterfactual trends, there were no differences in absolute reductions in the Theil index for caries-related extractions and tonsillectomy admission rates at 22 and 80 months after implementation (Table 3).

In sensitivity analysis including only the period after the change in ethnicity coding (April 2013 to December 2024), there were no differences in absolute or relative ethnic inequalities in admission rates for caries-related extractions or tonsillectomies at 22 and 80 months after implementation of the SDIL (Tables S2 and S3 and Fig. S2).

## Discussion

### Main findings of the study

This study found little evidence of an association between the implementation of the SDIL and reductions in ethnic inequalities in admission rates for caries-related extractions. Although relative reductions of 14% in the WMDR and 33% in the Theil index were observed at 80 months after SDIL implementation, the confidence intervals for these estimates were large and included zero. Over time, gaps in admission rates between Asian and other ethnic minority children and White children narrowed, while the direction of the inequality between black and white children reversed.

### What is already known about this topic

The SDIL reduced purchases and consumption of SSB, especially among high consumers such as low-income households and those with children.^25^^,^^26^ However, evidence on ethnic differences in SSB consumption is limited and inconsistent.^9^^,^^10^ The SDIL was associated with reductions in obesity prevalence among year-6 schoolgirls, but not among year-6 schoolboys or children in reception year. For year-6 schoolgirls, the largest reductions in prevalence of obesity were found in the two most deprived quintiles.^27^ A decrease in incidence rates for asthma among 5–18-year-olds was also observed after the SDIL, with similar reductions observed across deprivation quintiles.^28^

### What this study adds

Some explanations can be proposed for our findings. If high consumers are more common in certain ethnic groups, this could explain the narrowing of inequalities between Asian and other ethnic minority children and white children. Moreover, some SSB types not covered by the SDIL, such as milkshakes, flavoured milk, and milk-substitute drinks, may be preferred by some ethnic groups but not others, reflecting diverse cultural preferences and household practices. Another possibility is that the price elasticity of SSB varies across ethnic groups, as there is evidence that different ethnicities respond differently to changes in food and fast-food prices.^29^^,^^30^

Some methodological challenges encountered in this study could also explain the findings. Firstly, there was a sizeable amount of missing ethnicity data in HES.^31^^,^^32^ Ethnicity recording in NHS records may be less accurate for minority ethnic groups than the white population, and this accuracy can also vary by age, sex, region, and care pathway.^32^ This suggests that ethnicity data are missing not at random, which precludes the use of standard imputation techniques. Secondly, changes to NHS ethnicity coding were introduced in April 2013. This change resulted in a noticeable reduction in ethnic inequalities in admission rates. However, we found similar results in sensitivity analysis, excluding the period before the change in ethnicity coding. A third challenge relates to the categorisation of ethnicity and age. Ideally, admission rates would have been disaggregated by specific ethnic subgroups and age groups. However, this was not feasible due to small numbers in some categories, which limited statistical reliability. Aggregating data into broader ethnic and age groups increased statistical power but also masked known variations within these groups. A third challenge relates to ethnicity categorisation. A disaggregation of admission rates by ethnic subgroups would have been ideal. However, this was not possible given the small numbers for some subgroups. Analysing broad, rather than specific, ethnic groups increases statistical power but it also masks known variation between those specific groups. That said, the five broad ethnic categories used here correspond to those commonly used in government reports. A final challenge relates to sample size. Simulation-based power calculations for ITS analysis recommend that at least 72 data points are needed to detect a small effect size (0.5) in the change in outcome, assuming a statistical power of 0.80 and autocorrelation between −0.40 and − 0.90.^33^ However, these estimations apply to simple ITS with two segments (pre and post intervention periods), not to more complex models with multiple segments.

A final consideration is the potential role of confounding factors. Any concurrent changes in how dental services are organised, delivered, or funded in England could have acted as confounders, even though NHS dental care is free at the point of use for children. Because of this, variations in hospital admissions for caries-related extractions cannot be explained by clinical need alone. System-level factors are likely to play a role, including local availability of dental services, differences in care pathways (i.e. criteria for referral and admission), and the way hospitals are remunerated for care.^34^^,^^35^ Some of these structural and organisational features may also be associated with child ethnicity.

Taken together, our findings have important implications. They provide the first assessment of the SDIL’s distributional impact on child health, extending beyond area deprivation to include other key social dimensions, such as ethnicity. Understanding how policies reduce health inequalities is essential for prioritising those that most benefit disadvantaged groups. However, the lack of standardised ethnicity data in health records limits the ability to fully assess these impacts. Improving the completeness and consistency of ethnicity reporting across healthcare settings will be critical for future evaluations of the SDIL and for ensuring its continued effectiveness through regular monitoring.

### Limitations of this study

ITS analysis relies on the assumption that, in the absence of the intervention, the outcome would have followed its established trend, and that no other systematic changes or confounding events occurred at the same time as the intervention. Including a comparison group would have offered a stronger control for time-varying confounding. In the absence of an appropriate comparator, we used a negative control outcome that was not anticipated to be influenced by the SDIL. Although not ideal, the findings on tonsillectomy admission rates generally supported our hypothesis. Finally, we used hospital admissions for caries-related extractions as our primary outcome, which captures the most severe end of the caries distribution among children. Further assessment is needed to understand the impact of the SDIL on the entire distribution of caries.

## Conclusion

The implementation of the SDIL was not associated with reductions in ethnic inequalities in admission rates for caries-related extractions. Several explanations for these findings were discussed, with recommendations to improve ethnicity recording in health records.

## Supplementary Material

JPH_appendix_2025_12_02_Figure_S1_fdag016

JPH_appendix_2025_12_02_Figure_S2_fdag016

JPH_appendix_2025_12_02_Table_S1_fdag016

JPH_appendix_2025_12_02_Table_S2_fdag016

JPH_appendix_2025_12_02_Table_S3_fdag016

## Notes

**C.C. Salomon-Ibarra,** Research Associate in Dental Public Health

**J. Wu,** Professor of Biostatistics and Health Data Science

**V. Toffolutti,** Senior Lecturer in Health Economics

**E. Bernabe,** Professor of Dental Public Health
