# Supplementary material for: The impact of the UK soft drink industry levy on ethnic inequalities in admission rates for caries-related extractions
Source: J Public Health (Oxf). 2026 Feb 21;48(2):449–56. doi: 10.1093/pubmed/fdag016 (PMC13223591; doi:10.1093/pubmed/fdag016)
Supplement: JPH_appendix_2025_12_02_Figure_S1_fdag016 [file jph_appendix_2025_12_02_figure_s1_fdag016.pdf]

SUPPLEMENTARY FILE: FIGURE S1

Manuscript title:

The impact of the UK Soft Drink Industry Levy on ethnic inequalities in admission rates for caries-related extractions

Authors:

Salomon-Ibarra CC, Wu J, Toffolutti V, Bernabe E

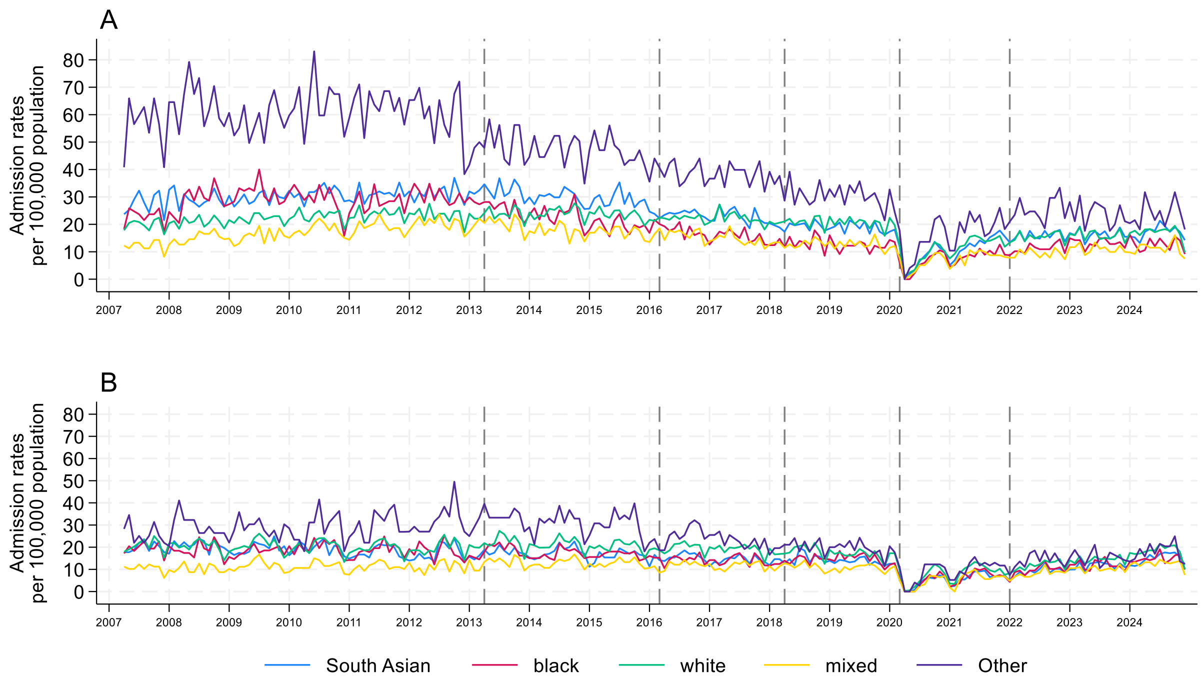

Figure S1. Hospital admission rates/100,000 population for caries-related extractions (panel A) and tonsillectomy (panel B) among 0-17-year-old children in England from April 2007 to December 2024. The five dashed vertical lines indicate the dates of change in ethnicity code (April 2013), SDIL announcement (March 2016), SDIL enforcement (April 2018), and start (March 2020) and end of lockdowns (January 2022).
