# Supplementary material for: The impact of the UK soft drink industry levy on ethnic inequalities in admission rates for caries-related extractions
Source: J Public Health (Oxf). 2026 Feb 21;48(2):449–56. doi: 10.1093/pubmed/fdag016 (PMC13223591; doi:10.1093/pubmed/fdag016)
Supplement: JPH_appendix_2025_12_02_Figure_S2_fdag016 [file jph_appendix_2025_12_02_figure_s2_fdag016.pdf]

SUPPLEMENTARY FILE: FIGURE S2

Manuscript title:

The impact of the UK Soft Drink Industry Levy on ethnic inequalities in admission rates for caries-related extractions

Authors:

Salomon-Ibarra CC, Wu J, Toffolutti V, Bernabe E

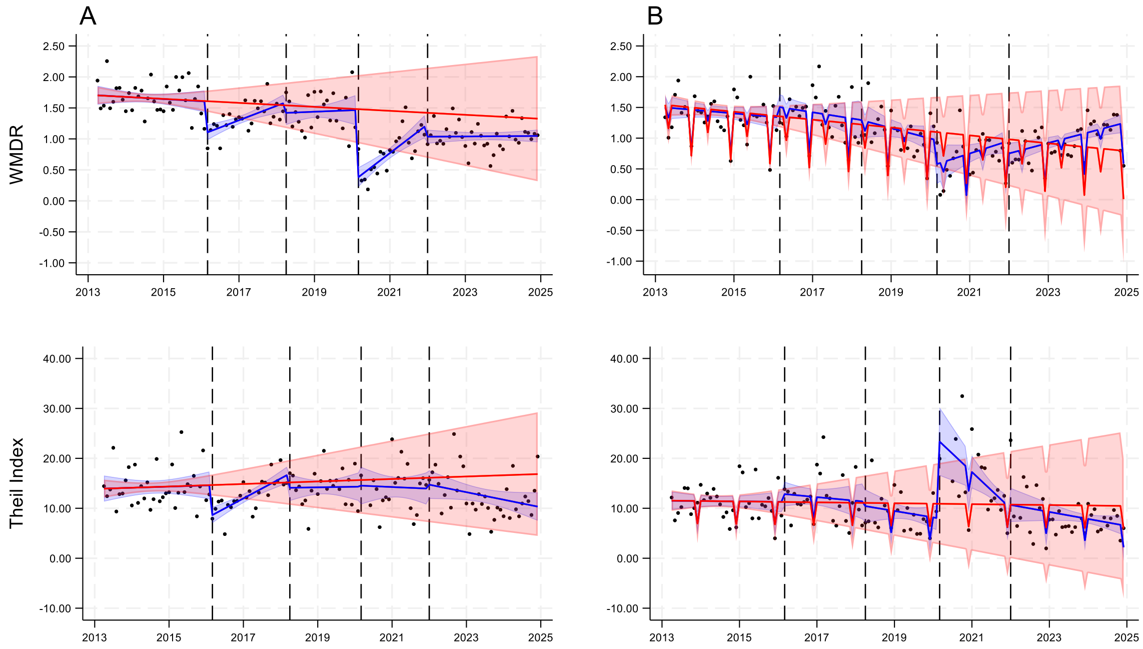

Figure S2. Absolute and relative ethnic inequalities in admission rates for caries-related extractions (panel A) and tonsillectomy (panel B) among 0-17-year-old children in England from April 2013 to December 2024. Absolute and relative inequalities were measured using the Weighted Mean Difference from the Reference group (WMDR) and the Theil index, respectively. The black dots show the observed data; the blue line indicates the predicted trend with 95%CI (blue shadow) fitted to the observed data and the red line indicates the counterfactual trend with 95%CI (red shadow) based on data from the pre-announcement period. The four dashed vertical lines indicate the dates of the SDIL announcement (March 2016) and enforcement (April 2018) as well as the start (March 2020) and end of lockdowns (January 2022).
