# Supplementary material for: The impact of the UK soft drink industry levy on ethnic inequalities in admission rates for caries-related extractions
Source: J Public Health (Oxf). 2026 Feb 21;48(2):449–56. doi: 10.1093/pubmed/fdag016 (PMC13223591; doi:10.1093/pubmed/fdag016)
Supplement: JPH_appendix_2025_12_02_Table_S1_fdag016 [file jph_appendix_2025_12_02_table_s1_fdag016.pdf]

SUPPLEMENTARY FILE: TABLE S1

Manuscript title:

The impact of the UK Soft Drink Industry Levy on ethnic inequalities in admission rates for caries-related extractions

Authors:

Salomon-Ibarra CC, Wu J, Toffolutti V, Bernabe E

Table S1. Categories included in each ethnic group.

Ethnic category	Codes from hospital records
South Asian	4: Indian<br>5: Pakistani<br>6: Bangladeshi<br>H: Indian (Asian or Asian British)<br>J: Pakistani (Asian or Asian British)<br>K: Bangladeshi (Asian or Asian British)<br>L: Any other Asian background
Black	1: Black - Caribbean<br>2: Black – African<br>3: Black – Other<br>M: Caribbean (Black or Black British)<br>N: African (Black or Black British)<br>P: Any other Black background
White	A: British (White)<br>B: Irish (White)<br>C: Any other White background
Mixed	D: White and Black Caribbean (Mixed)<br>E: White and Black African (Mixed)<br>F: White and Asian (Mixed)<br>G: Any other Mixed background
Other	7: Chinese<br>8: Any other ethnic group<br>R: Chinese (other ethnic group)<br>S: Any other ethnic group
Not known	9: Not given<br>99: Not known<br>99: Not known (2013 onwards)<br>Z: Not stated<br>X: Not known (prior 2013)
