# Supplementary material for: The impact of the UK soft drink industry levy on ethnic inequalities in admission rates for caries-related extractions
Source: J Public Health (Oxf). 2026 Feb 21;48(2):449–56. doi: 10.1093/pubmed/fdag016 (PMC13223591; doi:10.1093/pubmed/fdag016)
Supplement: JPH_appendix_2025_12_02_Table_S2_fdag016 [file jph_appendix_2025_12_02_table_s2_fdag016.pdf]

SUPPLEMENTARY FILE: TABLE S2

Manuscript title:

The impact of the UK Soft Drink Industry Levy on ethnic inequalities in admission rates for caries-related extractions

Authors:

Salomon-Ibarra CC, Wu J, Toffolutti V, Bernabe E

Table S2. Models for the impact of the soft drinks industry levy (SDIL) on absolute and relative inequalities in admission rates for caries-related extractions and tonsillectomies

	**WMDR**	**[95% CI]**	**Theil**	**[95% CI]**
Caries-related extractions				
New ethnicity coding period	1.70	[1.55, 1. 86]	13.95	[11.32, 16.58]
Time since new ethnicity coding	-0.003	[-0.01, 0.01]	0.02	[-0.12, 0.16]
Announcement period	-0.50	[-0.72, -0.27]	-5.96	[-9.30, -2.61]
Time since announcement	0.02	[0.01, 0.04]	0.31	[0.13, 0.49]
Enforcement period	-0.67	[-1.03, -0.30]	-8.80	[-13.73, -3.88]
Time since implementation	0.00	[-0.01, 0.02]	-0.01	[-0.27, 0.25]
Lockdown period	-1.75	[-2.29, -1.22]	-8.60	[-16.09, -1.12]
Time since lockdowns began	0.04	[0.03, 0.06]	-0.05	[-0.35, 0.26]
Recovery period	-1.95	[-2.56, -1.34]	-7.81	[-17.06, 1.45]
Time since recovery	0.003	[-0.01, 0.01]	0.14	[-0.34, 0.05]
Tonsillectomies				
New ethnicity coding period	13.95	[11.32, 16.58]	11.51	[9.67, 13.35]
Time since new ethnicity coding	0.02	[-0.12, 0.16]	-0.01	[-0.10, 0.09]
Announcement period	0.17	[-0.12, 0.46]	1.54	[-1.78, 4.87]
Time since announcement	0.00	[-0.03, 0.02]	-0.05	[-0.29, 0.18]
Enforcement period	0.16	[-0.41, 0.73]	0.65	[-6.04, 7.35]
Time since implementation	-0.01	[-0.03, 0.01]	-0.10	[-0.36, 0.15]
Lockdown period	-0.22	[-0.98, 0.54]	16.05	[5.36, 26.75]
Time since lockdowns began	-0.002	[0.00, 0.05]	-0.60	[-1.00, -0.21]
Recovery period	-0.43	[-1.28, 0.42]	16.76	[4.60, 28.92]
Time since recovery	0.02	[0.01, 0.03]	-0.11	[-0.29, 0.06]

WMDR: Weighted mean difference from the reference group.
A value of zero in the WMDR and Theil index indicate no inequality. Values greater than zero indicate higher admission rates in minority ethnic groups than the white group (reference).
