# Supplementary material for: The impact of the UK soft drink industry levy on ethnic inequalities in admission rates for caries-related extractions
Source: J Public Health (Oxf). 2026 Feb 21;48(2):449–56. doi: 10.1093/pubmed/fdag016 (PMC13223591; doi:10.1093/pubmed/fdag016)
Supplement: JPH_appendix_2025_12_02_Table_S3_fdag016 [file jph_appendix_2025_12_02_table_s3_fdag016.pdf]

**SUPPLEMENTARY FILE: TABLE S3**

**Manuscript title:**

The impact of the UK Soft Drink Industry Levy on ethnic inequalities in admission rates for caries-related extractions

**Authors:**

Salomon-Ibarra CC, Wu J, Toffolutti V, Bernabe E

**Table S3.** Predicted differences between the fitted and counterfactual trends for absolute and relative inequalities in caries-related extractions and tonsillectomies 22 and 80 months after the SDIL

| | **Absolute difference** | **[95% CI]** | **Relative difference (%)** | **[95% CI]** |
|---|---|---|---|---|
| *WMDR* | | | | |
| *Caries-related extractions* | | | | |
| At 22 months | -0.02 | [-0.61, 0.58] | -1.19 | [-40.86, 38.49] |
| At 80 months | -0.28 | [-1.30, 0.74] | -21.09 | [-81.66, 39.49] |
| *Tonsillectomies* | | | | |
| At 22 months | -0.12 | [-0.74, 0.49] | -11.26 | [-61.34, 38.83] |
| At 80 months[a] | 0.43 | [-0.47, 1.63] | 54.05[a] | [-150.36, 258.46] |
| *Theil index* | | | | |
| Caries-related extractions | | | | |
| At 22 months | -1.31 | [-8.53, 5.9] | -8.40 | [-51.28, 34.49] |
| At 80 months | -6.51 | [-19.21, 6.20] | -38.61 | [-86.75, 9.53] |
| *Tonsillectomies* | | | | |
| At 22 months | -2.85 | [-11.26, 5.56] | -26.12 | [-84.97, 32.73] |
| At 80 months | -3.76 | [-18.00, 10.48] | -63.11 | [-160.96, 160.96] |

Differences were estimated by subtracting the predicted values for the fitted trend (using the full time series) from the counterfactual trend (based on pre-announcement data only).
[a] This was the estimate for 79 months because the 80-month estimate was almost zero.
